# The Usefulness of Peripheral Blood Cell Counts to Distinguish COVID-19 from Dengue during Acute Infection

**DOI:** 10.3390/tropicalmed7020020

**Published:** 2022-01-30

**Authors:** Juan Fidel Osuna-Ramos, José Manuel Reyes-Ruiz, Luis Antonio Ochoa-Ramírez, Luis Adrián De Jesús-González, Rosalío Ramos-Payán, Carlos Noe Farfan-Morales, Alejandra Romero-Utrilla, Efrén Rafael Ríos-Burgueño, José Rodríguez-Millán, Rosa María del Ángel, Jesús Salvador Velarde-Félix

**Affiliations:** 1 Department of Infectomics and Molecular Pathogenesis, Center for Research and Advanced Studies (CINVESTAV-IPN), Ciudad de México 07360, Mexico; luis.dejesus@cinvestav.mx (L.A.D.J.-G.); carlos.farfan@cinvestav.mx (C.N.F.-M.); 2 Escuela de Medicina, Universidad Autónoma de Durango Campus Culiacán, Culiacán Rosales 80050, Mexico; 3 Unidad Médica de Alta Especialidad, Hospital de Especialidades No. 14, Centro Médico Nacional “Adolfo Ruiz Cortines”, Instituto Mexicano del Seguro Social (IMSS), Veracruz 91897, Mexico; jose.reyesr@imss.gob.mx; 4 “Bernardo J Gastélum”, Servicios de Salud de Sinaloa, Hospital General de Culiacán, Culiacán Rosales 80030, Mexico; luisochoaram@hotmail.com (L.A.O.-R.); rael_rios@yahoo.com.mx (E.R.R.-B.); fidel_40@hotmail.com (J.R.-M.); 5 Facultad de Ciencias Químico-Biológicas, Universidad Autónoma de Sinaloa, Culiacán Rosales 80040, Mexico; rosaliorp@uas.edu.mx; 6 Departamento de Anatomía Patológica, Instituto Mexicano del Seguro Social (IMSS), Culiacán Rosales 80220, Mexico; alejandraromeroutrilla@gmail.com; 7 Departamento de Anatomía Patológica, Hospital Civil de Culiacán, Centro de Investigación y Docencia en Ciencias de la Salud, Universidad Autónoma de Sinaloa, Culiacán Rosales 80030, Mexico; 8 Escuela de Biología, Universidad Autónoma de Sinaloa, Culiacán Rosales 80040, Mexico

**Keywords:** COVID-19, dengue, peripheral blood cells count neutrophils, neutrophil–lymphocyte ratio, predictors

## Abstract

COVID-19 and dengue disease are challenging to tell apart because they have similarities in clinical and laboratory features during the acute phase of infection, leading to misdiagnosis and delayed treatment. The present study evaluated peripheral blood cell count accuracy to distinguish COVID-19 non-critical patients from non-severe dengue cases between the second and eleventh day after symptom onset. A total of 288 patients infected with SARS-CoV-2 (n = 105) or dengue virus (n = 183) were included in this study. Neutrophil, platelet, and lymphocyte counts were used to calculate the neutrophil–lymphocyte ratio (NLR), the platelet–lymphocyte ratio (PLR), and the neutrophil–lymphocyte*platelet ratio (NLPR). The logistic regression and ROC curves analysis revealed that neutrophil and platelet counts, NLR, LPR, and NLPR were higher in COVID-19 than dengue. The multivariate predictive model showed that the neutrophils, platelets, and NLPR were independently associated with COVID-19 with a good fit predictive value (*p* = 0.1041). The neutrophil (AUC = 0.95, 95% CI = 0.84–0.91), platelet (AUC = 0.89, 95% CI = 0.85–0.93) counts, and NLR (AUC = 0.88, 95% CI = 0.84–0.91) were able to discriminate COVID-19 from dengue with high sensitivity and specificity values (above 80%). Finally, based on predicted probabilities on combining neutrophils and platelets with NLR or NLPR, the adjusted AUC was 0.97 (95% CI = 0.94–0.98) to differentiate COVID-19 from dengue during the acute phase of infection with outstanding accuracy. These findings might suggest that the neutrophil, platelet counts, and NLR or NLPR provide a quick and cost-effective way to distinguish between dengue and COVID-19 in the context of co-epidemics in low-income tropical regions.

## 1. Introduction

Coronavirus disease 2019 (COVID-19), caused by severe acute respiratory syndrome coronavirus 2 (SARS-CoV-2), is a public health emergency affecting more than 200 countries worldwide [1]. COVID-19 is classified according to the severity of the symptoms: mild, moderate, severe, and critical, leading to death [2]. Nowadays, the COVID-19 pandemic continues to spread worldwide, including subtropical and tropical regions where endemic diseases, such as dengue, are part of continuing public health surveillance [3,4,5]. Dengue virus (DENV) is the etiological agent of dengue disease classified into non-severe and severe dengue, with fatal complications in the severe cases [3]. Patients with dengue or COVID-19 develop symptoms (within 4 to 10 days; incubation period), including fever, headache, rash, myalgia, arthralgia, diarrhea, nausea, and vomiting [6,7,8]. Due to the co-circulation of DENV and SARS-CoV-2 and similar clinical manifestations that both illnesses share, a substantial clinical misdiagnosis should be considered during the first 10 days post-viral infection [4,9,10,11]. Therefore, the patients from dengue-endemic regions with confirmed cases of COVID-19 must be explored for both diseases.

Interestingly, misdiagnoses between dengue and COVID-19, based on clinical manifestations have been reported in patients with a skin rash and thrombocytopenia [12,13]. Initially, these patients from Thailand were diagnosed with dengue but were later diagnosed with COVID-19 after developing respiratory symptoms and when the final diagnosis of SARS-CoV-2 infection was confirmed by reverse transcription polymerase chain Reaction (RT-PCR) [12,13]. In addition to the similar clinical presentations of dengue and COVID-19, misdiagnosis may be due to serological cross-reactivity between DENV and SARS-CoV-2. Yan et al. reported that patients from Singapore were initially positive for dengue IgM and IgG rapid serological testing [10]. However, these patients had worsening cough and dyspnea, and they were positive for SARS-CoV-2 but negative for DENV by RT-PCR, so that the initial anti-dengue IgM/IgG test was deemed to have been a false positive [10]. Moreover, other patients had false positive dengue serology, who were later confirmed to have COVID-19 during a dual outbreak of SARS-CoV-2 and DENV [14]. Thus, there remains the possibility that there is SARS-CoV-2 infection among dengue-diagnosed patients. To solve this problem, it is necessary to detect and differentiate cases of dengue and COVID-19 and there is an urgent need for a diagnostic method to prevent misdiagnosis [7,15]. In this regard, tools with higher diagnostic accuracy that might help to improve patient outcomes are required.

Although both viruses share similar hematological and biochemical findings, such as leucopenia and thrombocytopenia, these data may provide vital information to assess the severity of the disease [16,17,18,19,20,21,22]. The absolute neutrophil (NEU) and platelet (PLT) count, the neutrophil–lymphocyte (LYM) ratio (NLR), and the platelet–lymphocyte ratio (PLR) are standard laboratory tests, and they are good predictors for cases of critical COVID-19 [22,23,24,25,26]. Hence, we hypothesized that these parameters obtained from the peripheral blood cells count could distinguish patients with non-critical COVID-19 from non-severe dengue cases.

## 2. Materials and Methods

### 2.1. Study Design and Participants

We conducted a retrospective and cross-sectional study at the Culiacan General Hospital, Sinaloa, Mexico. A total of 105 COVID-19 patients (all Mestizos from Northwestern Mexico) with a positive nucleic acid test for SARS-CoV-2 from March to April 2020 were included. The clinical sample collection, processing, and SARS-CoV-2 testing for all patients were based on WHO guidelines [2]. COVID-19 patients who met any of the following criteria were excluded from the study: (1) patients with previous DENV infection or other flaviviruses; (2) individuals with SARS-CoV-2/DENV coinfection; (3) patients aged ≤ 18 years; (4) pregnant patients; (5) patients with chronic renal dysfunction, malignant tumor, liver cirrhosis, acquired immune deficiency, and other autoimmune and inflammatory diseases. The Institutional Ethics and Research Committee approved the study at the Culiacan General Hospital, Sinaloa, Mexico.

Disease severity was defined according to the “Diagnosis and Treatment Protocol for Novel Coronavirus Pneumonia” [27]. Patients with a confirmed diagnosis of COVID-19 were classified into four types: (1) mild—patients with slight clinical symptoms and no imaging finding of pneumonia; (2) moderate—patients with fever and respiratory symptoms, and signs of pneumonia on radiologic assessment; (3) severe—patients meeting any of the following criteria: (a) shortness of breath, respiratory rate ≥ 30 times/min; (b) oxygen saturation ≤93% at rest; (c) partial pressure of oxygen/fraction of inspired oxygen ≤ 300 mmHg; (d) pulmonary imaging showing the significant progression of lesion > 50% within 24 to 48 h; and (4) critical—patients showing any of the following conditions: respiratory failure requires mechanical ventilation, shock, combined with other organ failure requires intensive care and treatment). For further analysis in this study, the patients classified as mild, moderate, and severe were grouped as “non-critical”.

Regarding dengue disease, data from patients (all patients from northwestern Mexico) with confirmed dengue were extracted from a cohort study conducted at Culiacan General Hospital during a dengue outbreak, before the COVID-19 pandemic [28]. Dengue patients who met any of the following criteria were excluded from the study: (1) patients with previous DENV infection or other arbovirus infections; (2) children or patients aged ≤ 18 years; (3) pregnant patients; (4) patients having chronic renal dysfunction, malignant tumor, liver cirrhosis, acquired immune deficiency, and other autoimmune and inflammatory diseases. All patients in the study were clinically diagnosed and treated according to the 2009 WHO dengue guideline as non-severe and severe dengue. The acute dengue infection was confirmed by the SD BIOLINE dengue duo rapid test (NS1 Ag and immunoglobulins IgM/IgG) [29]. The non-severe cases of DENV infection were used to perform an appropriate comparison with SARS-CoV-2-infected patients.

### 2.2. Data Collection

All data from the patients meeting the inclusion criteria were abstracted from the electronic medical records. Clinical parameter included age, sex, symptoms, and laboratory results were collected on admission (between second and eleventh day after symptom onset) for both COVID-19 and dengue.

### 2.3. Definitions

The neutrophil–lymphocyte ratio (NLR) is defined as the product between blood neutrophil (NEU) and lymphocyte (LYM) counts—NLR = NEU/LYM [23]. Platelet–lymphocyte ratio (PLR) was calculated by dividing the total absolute platelet (PLT) counts over total lymphocyte counts—PLR = PLT/LYM [23]). Finally, neutrophil–lymphocyte*platelet ratio (NLPR) was calculated using the following formula: NLPR = NEU/(LYM)(PLT) [23,30]. 

### 2.4. Statistical Analysis

Categorical data were represented in numbers and frequencies (%), and continuous variables were expressed as median and interquartile ranges (IQR). The Pearson’s chi-square test was used to compare categorical variables, and the Mann–Whitney U test was used to analyze continuous variables between COVID-19 and dengue disease. Gender and age-adjusted logistic regression was performed to calculate coefficients, adjusted odds ratios (OR), and 95% confidence intervals (95% CI). The goodness of fit model was evaluated using the Hosmer–Lemeshow test. Receiver operating characteristic (ROC) curves and area under the curves (AUC) were calculated to determine the predictive accuracy for differentiating between COVID-19 and dengue disease of peripheral blood leucocyte counts and ratios in the early disease phase. The De Long et al. [31] method was used for calculating standard errors (SE); exact binomial 95% CI for the AUC and sensitivity and specificity with 95% CI values were determined based on the best cut-off point obtained with the Youden index. The MedCalc software version 19 (MedCalc, Ostend, Belgium), R version 4.1.0 and Rstudio version 1.3 (R & Rstudio, Boston, MA, USA) were used for the statistical analysis and statistical significance was set with a two-tailed *p*-value < 0.05.

### 2.5. Ethics Approval

This study was approved by the Institutional Ethics and Research Committee from the Culiacan General Hospital. Including the exemption of the requirement for informed consent. The study was compliant with the Declaration of Helsinki. 

## 3. Results

The demographic characteristics and the clinical data of the patients with COVID-19 (n = 105) or dengue (n = 183) are described in Table 1. Significant differences were observed in the gender and age among COVID-19 non-critical and dengue non-severe patients. Males (61%) were more affected in the COVID-19 group, while in the dengue group, it was females (67%) (*p* ≤ 0.001). Regarding the age difference, the median age was 56 and 33 years for COVID-19 and dengue groups, respectively (*p* < 0.001) (Table 1). The overall median day after symptoms onset was four days, and it was seven and four days for COVID-19 and dengue patients, respectively (Table 1). According to the reported clinical data, fever was found to be a manifestation reported with a high frequency (over 80%) and headache in more than 70% of the cases of both COVID-19 and dengue during acute infection (Table 1). On the other hand, although most dengue patients reported myalgias and arthralgias, more than half of the patients with COVID-19 also manifested these symptoms (Table 1).

**Figure 1:**
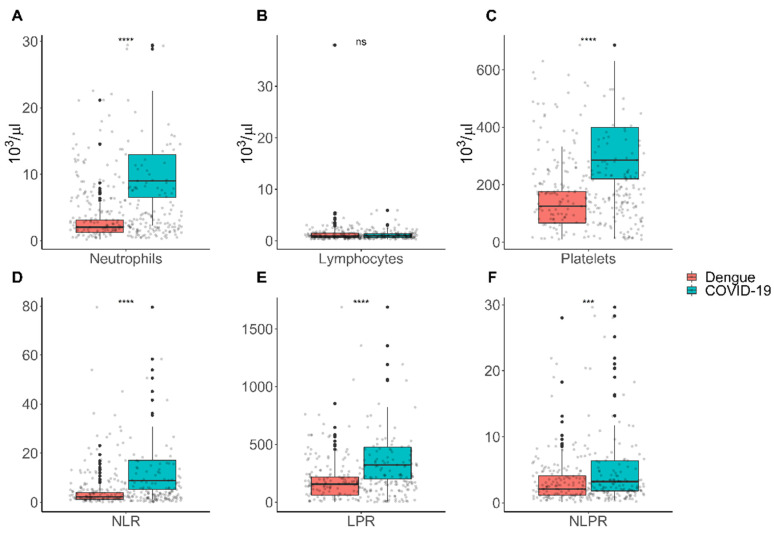
Hematological parameter and ratios for COVID-19 and dengue during the acute infection. (**A**) Neutrophils; (**B**) lymphocytes; (**C**) platelets; (**D**) neutrophil–lymphocyte ratio (NLR); (**E**) platelet–lymphocyte ratio (PLR); (**F**) neutrophil–lymphocyte*platelet ratio (NLPR). Normal Values: neutrophils: 1.5–7.0 × 10^3^/μL; platelets: 150–450 × 10^3^/μL; lymphocytes: 1.0–4.2 × 10^3^/μL. The p-value was obtained using Mann–Whitney U test. The following convention was used for symbols indicating statistical significance: ns: *p* > 0.05; * *p* ≤ 0.05; ** *p* ≤ 0.01; *** *p* ≤ 0.001; **** *p* ≤ 0.0001.

Concerning the hematological parameter, neutrophils (9 × 10^3^/µL (IQR = 6.5) versus 2 × 10^3^/µL (IQR = 1.9)) and platelets (286 × 10^3^/µL (IQR = 179) vs. 125 × 10^3^/µL (IQR = 109)) counts were significantly higher in patients with COVID-19 than dengue patients (*p* < 0.001) (Figure 1). Nonetheless, the lymphocyte (1 × 10^3^/µL (IQR = 0.8) versus 0.9 × 10^3^/µL (0.9)) level count showed no significant difference between COVID-19 and dengue disease (Figure 1A–C). Regarding leucocyte ratios, the NLR (8.8 (IQR = 11.8) versus, 2.1 (IQR = 2.7)), PLR (323 (IQR = 276) versus 155 (IQR = 157)), and PNLR (3.2 (IQR = 4.5) versus 2.1 (IQR = 2.9)) levels were significantly higher in patients with COVID-19 than patients with dengue (*p* < 0.001) (Figure 1D–F). 

The following process was carried out to find an adequate predictive model to differentiate between COVID-19 and dengue disease. The variables of hematological parameters and leucocyte ratios were analyzed by logistic regression. Due to the statistically significant differences found for both groups’ disease in demographic characteristics, models were adjusted for age and gender for the goodness of fit. In the univariate predictive model, all hematological parameters and ratios were evaluated, platelets, neutrophils, NLR, and PLR were independently associated with COVID-19 (Table 2). However, in the multivariate predictive model, neutrophils, platelets, and NLPR were independently associated with COVID-19, showed a good fit predictive value (*p* = 0.1041) (Table 2). As a part of classification model evaluation, the percentages of COVID-19 cases correctly classified using neutrophils, platelets, and NLPR as predictive parameters were 89.93%, 88.89%, and 83.72%. Based on logistic regression multivariate analysis, the percentage to adequately classify COVID-19 patients using the significative hematological parameters platelets, neutrophil counts with the NLR, and NLPR was 92.01% to distinguish COVID-19 from dengue patients during the acute phase of infection.

To determine the accuracy of hematological parameters and leucocyte ratios to differentiate between COVID-19 and dengue during the acute phase of infection, ROC curves and AUC were calculated, the values were described in Table 3. 

**Figure 2:**
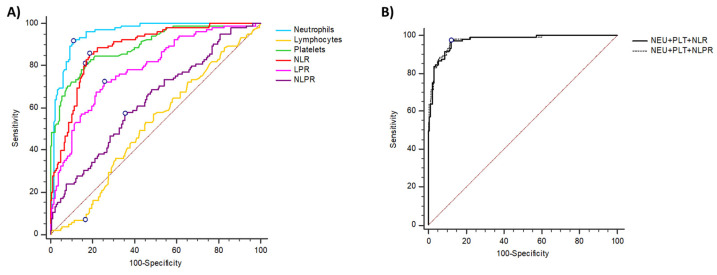
ROC curves, AUC for COVID-19, and dengue hematological parameters and ratios. (**A**) ROC values of neutrophils, lymphocytes, platelets, NLR, PLR, and NLPR for prediction between COVID-19 and dengue disease. The mark point in the line corresponds to Youden’s index best cut-off point. (**B**) Predicted probabilities on combining neutrophils and platelets with NLR or NLPR, the adjusted AUC was calculated based on the logistic regression analysis model.

The best predictive value for COVID-19 was neutrophils as it showed the highest sensitivity and specificity (Figure 2 and Table 3). On the other hand, the NLR showed the best AUC predictive value and high sensitivity and specificity values (Figure 2A and Table 3). Remarkably, based on predicted probabilities on combining neutrophils and platelets with NLR or NLPR, the adjusted AUC was 0.97 (95% CI = 0.94–0.98) to differentiate COVID-19 from dengue (Figure 2B).

In summary, these findings suggested that neutrophils and combination of neutrophils, NLR, NLPR, and platelet counts can be used to distinguish COVID-19 from dengue during acute infection.

## 4. Discussion

During acute infection by DENV or SARS-CoV-2, the patients develop diarrhea, fever, headache, rash, arthralgia, myalgia, nausea, and vomiting [6,7,8]. Lower respiratory symptoms could help distinguish between patients infected with SARS-CoV-2 and DENV. However, this clinical manifestation occurs approximately 7 days after the onset of symptoms, along with the risk of developing hypoxemic respiratory failure severe enough to require intubation [31]. Moreover, a misdiagnosis due to serological cross-reactivity between DENV and SARS-CoV-2 infections has been reported in the first 7 days after the onset of symptoms [10,12,13]. Therefore, it is necessary detect and differentiate the patients with COVID-19 from the cases of dengue in a timely manner to improve patient outcomes. In this regard, this study was performed with patients diagnosed with COVID-19 or dengue between the second and eleventh days after symptom onset. The differences found in the clinical data collected provided us with the basis for judging whether clinical equipoise was present. Based on the available information, we found that both fever and headache were manifestations reported with a high frequency in both COVID-19 and dengue during acute infection. Both symptoms are important for the clinical diagnosis of both diseases, which creates a dilemma when making clinical diagnostic decisions [7].

Earlier differentiation of patients with dengue or COVID-19 based on routine laboratory changes will give its widespread COVID-19 concurrence with other infectious diseases, such as dengue, that can occur especially in subtropical and tropical regions where dengue disease is highly prevalent [4,5]. Due to the tropical climate and the spread of SARS-CoV-2 in Mexico and other endemic areas, individuals are at risk for coinfection with dengue. Recently, several case reports have described similar clinical manifestations in patients coinfected with COVID-19 and dengue [32,33,34]. The aforementioned represents a challenge in health care because dengue and COVID-19 are difficult to distinguish as they have shared some clinical and laboratory features, especially in the early phase of disease [9,10]. Therefore, cheap, rapid, sensitive, accurate, and accessible tools to distinguish between dengue and COVID-19 are essentials, particularly in low-income countries where large-scale RT-PCR screening diagnosis is not available [4,9,34].

The present study evaluated the predictive accuracy of hematological parameters and identified differences between COVID-19 and dengue disease during the acute phase of infection, based on inexpensive blood count cell data. We found that neutrophil, platelet, and leucocyte ratios were higher in patients with COVID-19 than in dengue patients. Of these parameters, the neutrophil and platelet count and NLR levels can differentiate between COVID-19 and dengue with adequate accuracy. 

Recently, a cross-sectional study from Colombia reported hematological differences between COVID-19 and dengue patients [35,36]. They observed that differences between dengue and COVID-19 patients regarding platelet and neutrophil count. Additionally, they observed that the NLR was significantly higher in COVID-19 patients than in dengue patients, showing that the predictive ability of NLR had an AUC of 77%, a sensitivity of 100%, and a specificity of 54% in the classification between dengue and COVID-19 [36]. The value for AUC, found by these authors (0.7–0.8), is considered acceptable as a predictor [37]. We supported the proposal presented in the present study to combine parameters as neutrophils, platelets, and NLR, to obtain excellent predictive values to distinguish between COVID-19 and dengue disease. We found higher specificity and sensitivity values and AUC of 96% and 91% for neutrophils and NLR, respectively, and depending on the neutrophils and platelets with NLR values, the AUC accuracy for COVID-19 increased to 97% to differentiate from dengue.

Neutrophils’ role during SARS-CoV-2 infection has attracted research attention because they may be playing critical roles during the pathogenesis of COVID-19 disease [38,39,40]. Consequently, investigations in patients with COVID-19 have reported hematological and immunological alterations [39,41,42,43]. In particular, the increased number of circulating neutrophils and NLR elevation has been observed [26,39,44,45].

The role of hematological parameters to differentiate COVID-19 from other infections disease has been previously studied [46]. In this regard, Lin et al. reported the impact of monocyte distribution width (MDW) and NLR for distinguishing COVID-19 and influenza from other upper respiratory tract diseases [46]. They found that the AUC of MDW and NLR for distinguishing COVID-19 from other upper respiratory tract infections (URIs) were 0.703 and 0.673, respectively. Besides, they stated that the combination of MDW and NLR can distinguish COVID-19 from influenza, and URIs effectively showed an AUC of 0.705 [46]. It is possible that in the study mentioned above, the fact of comparing 2 viral diseases of the respiratory tract, with a small number of COVID-19 patients included (n = 9), could have been the reason why they did not identify significant differences in other hematological parameters.

Neutrophilia has been observed in patients with COVID-19; it occurs in 6 of 8 patients, from 7 to 19 days after the onset of symptoms [47]. In the present study, we included patients in the acute phase of the infection for both diseases (between the 5th and 11th day after symptoms). Neutrophilia (9.40 × 10^3^/μL (IQR: 6.90–12.99 × 10^3^/μL)) was observed only in COVID-19 patients (Table 1). On the other hand, the level of lymphocytes was similar between the two infections. However, it was found in lower levels than the standard value (1.0–4.2 × 10^3^/μL) for both disease groups.

The neutrophil–lymphocyte ratio (NLR) is a simple parameter that quickly assesses the inflammatory status [48]. NLR has been extensively studied in many infectious diseases. It has been identified that the NLR in COVID-19 may be a proper prognostic parameter [26,44,49,50]. Our study found that among hematologic ratios, the NLR was elevated in COVID-19 patients compared with dengue patients. Although the AUC value was lower than the hematologic parameters alone, it is within acceptable ranges for distinguishing between COVID-19 and dengue.

Dengue is a systemic disease in which leucopenia and thrombocytopenia have been identified as common hematological disorders due to the endothelium’s involvement, leading to excessive plasma leakage due to the immunological response [29,51]. On the other hand, it has been observed that most patients with severe coronavirus disease have presented thrombocytopenia and significant evidence suggests that SARS-CoV-2 virions interact directly with platelets to trigger activation leading to thrombocytopenia and thrombosis [52]. In addition, thrombocytopenia in COVID-19 has been described as common and is associated with increased mortality in hospitalized patients [53]. So far, two meta-analyses have presented that thrombocytopenia reflected a worse outcome in patients with COVID-19 [54,55]. However, these studies’ pooled ORs showed a wide range of CI and a wide heterogeneity [56]. In the present study, we found platelet count levels (279 × 10^3^/μL (IQR: 205–374 × 10^3^/μL)) within average values (150–450 × 10^3^/μL) in the group of COVID-19 patients; however, thrombocytopenia was detected only in the group of patients with dengue (110 × 10^3^/μL (IQR: 43–170 × 10^3^/μL)). The fact that our study has included for analysis and comparison cases of COVID-19 in an early phase in order to compare with dengue shows that platelet levels in the early phase of COVID-19 were normal, and these levels could be altered as the disease evolves and most likely are associated with severe manifestations such as sepsis [52,53]. An existing concern is whether SARS-CoV-2 interacting directly with platelets could lead to antibody-dependent enhancement (ADE). This is unlikely, because the disease caused by SARS-CoV-2 lacks the clinical, epidemiological, biological, or pathological attributes of ADE disease exemplified by dengue viruses [57]. The evidence that SARS-CoV-2 can interact with platelets is strong. Such interaction of pathogens with platelets is mediated by direct interaction of a pathogen surface protein with a platelet receptor or by binding a pathogen-bound antibody to FcγRIIa [52]. FcγRIIA and FcγRIIIA receptors have recently been demonstrated to mediate a modest ADE of infection against SARS-CoV-2. Although an ADE of infection was observed in macrophages derived from monocytes infected with SARS-CoV-2, including its variants, the expression of proinflammatory cytokines/chemokines was not increased in macrophages. Thus, these antibodies do not contribute to excessive cytokine production by macrophages [58]. The preceding description reinforces the idea of ruling out a possible ADE in COVID-19, since unlike infections by some of the DENV viruses, SARS-CoV-2 predominantly infects the respiratory epithelium, not macrophages [57].

Our study’s other differences were concerning age and gender, with COVID-19 patients ranging from 44 to 67 years, while dengue patients ranged from 25 to 46 years. Furthermore, more males were affected in the COVID-19 group, and more females were involved in the dengue group. Despite these differences, we found adequate goodness of fit when the prediction model was adjusted by age and gender. Finding platelets, neutrophils, and NLR were hematological parameters independently associated with COVID-19 and in combination the AUC was 0.97, which is considered an outstanding predictive value, suggesting these parameters could have a high clinical utility to differentiate these diseases.

Due to our study’s design and to avoid bias of clinical information, we were limited to analyzing only hematological parameters found in each patient’s clinical records. In addition, we consider it an advantage that the diagnosis of dengue cases was carried out before the COVID-19 emerged, which eliminates errors due to case selection bias. However, we found statistically significant differences and good predictive values. This study could lead to further studies, including a validation cohort to validate the observations made, because the design of this study could not allow for this. On the other hand, the model’s performance was evaluated, estimating the model’s prediction accuracy based on logistic regression analysis. It was found that for a model combining the hematologic values of neutrophil, platelet, and NLR or NLPR, the prediction for COVID was above 95% and has high sensitivity and specificity values. Here, we report an economical and efficient option to differentiate between COVID-19 and dengue, practically applied in low-income tropical regions where the syndemic scenario is a significant healthcare concern.

Furthermore, this study was conducted with patients with non-severe dengue and non-critical COVID-19. Therefore, more studies are required to analyze cases with severe dengue and critical COVID-19 to know if the values of the hematological predictors are different and to distinguish between these infections.

## Figures and Tables

**Table 1:** Demographic and reported clinical data of COVID-19 non-critical and dengue non-severe patients.

Characteristic	Overall, N = 288 *	COVID-19, n = 105 *	Dengue, n = 183 *	*p*-Value
Gender				<0.001
Female	163 (57)	41 (39)	122 (67)	
Male	125 (43)	64 (61)	61 (33)	
Age	40.0 (27.2)	56.0 (22.0)	33.0 (21.0)	<0.001
Severity				
Non-Critical	105 (36)	105 (100)	0 (0)	<0.001
Non-Severe	183 (64)	0 (0)	183 (100)	
Day after Symptom Onset	4 (3)	7 (5.5)	4 (2)	<0.001
Fever	267 (93)	85 (81)	182 (99)	<0.001
Headache	210 (73)	76 (72)	134 (73)	ns
Myalgya	218 (76)	57 (54)	161 (88)	<0.001
Arthralgia	228 (79)	59 (56)	169 (92)	<0.001

* number (%) for qualitative data; median and interquartile ranges (IQR) for quantitative data. The *p*-value was obtained using Pearson’s chi-square test for categorical variables and Mann–Whitney U test for continuous variables.

**Table 2:** Univariate and multivariate regression logistic analysis of COVID-19 and dengue disease and peripheral blood cell count adjusted by age, gender, and severity.

	Univariate				Multivariate			
	Coeff	OR	95% CI	*p*-value	Coeff	OR	95% CI	*p*-Value
**Neutrophils**	0.54	1.72	1.50–1.99	<0.0001	0.27	1.31	1.03–1.67	0.0260
**Lymphocytes**	−0.07	0.93	0.79–1.09	0.3858	−0.20	0.81	0.39–1.72	0.5904
**Platelets**	0.01	1.01	1.01–1.02	<0.0001	0.02	1.02	1.01–1.03	<0.0001
**NLR**	0.22	1.25	1.166–1.34	<0.0001	−0.025	0.97	0.80–1.19	0.8051
**PLR**	0.006	1.00	1.00–1.00	<0.001	−0.002	0.99	0.99–1.00	0.3579
**NLPR**	0.05	1.05	0.99–1.13	0.0895	0.192	1.21	1.05–1.39	0.0074

Coefficient (Coeff); odds ratios (OR); 95% confidence intervals (95% CI). Neutrophil–lymphocyte ratio (NLR); platelet–lymphocyte ratio (PLR); neutrophil–lymphocyte*platelet ratio (NLPR). The goodness of fit multivariate logistic regression model was evaluated using the Hosmer–Lemeshow test, showing the respective *p*-value = 0.4657.

**Table 3:** Area under the ROC curve (AUC) and criterion values for hematologic parameters and leucocyte ratios in COVID-19 and dengue patients.

	AUC	95% CI	Cut-Off Point	*p*-Value	Sens %	Spec %
**Neutrophils**	0.95	0.92–0.97	4.39	<0.0001	91.4	89.1
**Lymphocytes**	0.51	0.45–0.57	2.14	0.6740	6.7	83.6
**Platelets**	0.89	0.85–0.93	198	<0.0001	81	83.6
**NLR**	0.88	0.84–0.91	4.42	<0.0001	85.71	81.42
**PLR**	0.78	0.73–0.83	213.46	<0.0001	72.4	74.3
**NLPR**	0.63	0.57–0.69	2.91	0.0001	57.14	64.48

Area under the curve (AUC); 95% confidence interval (95% CI); sensitivity % (Sens %); specificity % (Spec %). Cut-off points were calculated based on the Youden index and *p*-value for AUC were determined by the De Long method.

## Data Availability

The datasets generated and analyzed during the study are available from the corresponding authors upon reasonable request.
